# Understanding public perceptions and discussions on diseases involving chronic pain through social media: cross-sectional infodemiology study

**DOI:** 10.1186/s12891-024-07687-5

**Published:** 2024-07-22

**Authors:** M. T. Valades, M. Montero-Torres, F. J. Lara-Abelenda, F. Carabot, M. A. Ortega, M. Álvarez-Mon, M. A. Alvarez-Mon

**Affiliations:** 1 Department of Medicine and Medical Specialties, Faculty of Medicine and Health Sciences, University of Alcala, Alcala de Henares, Madrid, Spain; 2 Ramon y Cajal Institute of Sanitary Research (IRYCIS), Ramon y Cajal Hospital, Madrid, Spain; 3 Department of Signal Theory and Communications, Telematics and Computing Systems, Rey Juan Carlos University, Madrid, Spain; 4 Immune System Diseases-Rheumatology and Internal Medicine Service, Center for Biomedical Research in Hepatic and Digestive Diseases Network, University Hospital Principe de Asturias, Alcala de Henares, Madrid, Spain; 5 Department of Psychiatry and Mental Health, University Hospital Infanta Leonor, Madrid, Spain; 6 CIBERSAM-ISCIII, Biomedical Research Networking Centre in Mental Health, Madrid, Spain

**Keywords:** Social Media, Fibromyalgia, Headache, Paraplegia, Multiple Sclerosis, Neuralgia, Chronic Pain, Infodemiology

## Abstract

**Background:**

Chronic pain is a highly prevalent medical condition that negatively impacts quality of life and is associated with considerable functional disability. Certain diseases, such as fibromyalgia, headache, paraplegia, neuropathy, and multiple sclerosis, manifest with chronic pain.

**Objective:**

The aim of this study is to examine the number and type of tweets (original or retweet) related to chronic pain, as well as to analyze the emotions and compare the societal impact of the diseases under study.

**Methods:**

We investigated tweets posted between January 1, 2018, and December 31, 2022, by Twitter users in English and Spanish, as well as the generated retweets. Additionally, emotions were extracted from these tweets and their diffusion was analyzed. Furthermore, the topics most frequently discussed by users were collected.

**Results:**

A total of 72,874 tweets were analyzed, including 44,467 in English and 28,407 in Spanish. Paraplegia represented 23.3% with 16,461 of the classified tweets, followed by headache and fibromyalgia with 15,337 (21.7%) and 15,179 (21.5%) tweets, respectively. Multiple sclerosis generated 14,781 tweets (21%), and the fewest tweets were related to neuropathy with 8,830 tweets (12.5%). The results showed that the primary emotions extracted were "fear" and "sadness." Additionally, the reach and impact of these tweets were investigated through the generated retweets, with those related to headaches showing the highest interest and interaction among users.

**Conclusion:**

Our results underscore the potential of leveraging social media for a better understanding of patients suffering from chronic pain and its impact on society. Among the most frequently encountered topics are those related to treatment, symptoms, or causes of the disease. Therefore, it is relevant to inform the patient to prevent misconceptions regarding their illness.

**Supplementary Information:**

The online version contains supplementary material available at 10.1186/s12891-024-07687-5.

## Background

The International Association for the Study of Pain (IASP) defines chronic pain as "an unpleasant sensory and emotional experience associated with, or resembling that associated with, actual or potential tissue damage"[1, 2]. It is a highly prevalent medical condition, affecting 20% of the global population[3], negatively impacting the quality of life[4], and is associated with considerable functional disability[5]. Consequently, current efforts to diagnose and identify diseases associated with chronic pain are becoming increasingly notable.

Some neurologically affecting diseases manifest with chronic pain. Moreover, their prevalence is increasing globally[6], making them a frequent reason for consultations. Five examples of these diseases are fibromyalgia, headaches, paraplegia, neuropathy, and multiple sclerosis.

For instance, fibromyalgia is characterized by widespread chronic pain, physical exhaustion, cognitive difficulties, depressed mood, sleep problems, and impairment of health-related quality of life[7]. The most common neurological disorder in the population is headache[8, 9], which has a significant negative impact on individuals, causing stress, fatigue, anxiety, and irritability[8]. Chronic headache is defined as a headache occurring at least 50% of the day for at least 3 months, lasting at least 2 h per day[10]. In the case of paraplegia, chronic pain often presents in individuals with spinal cord injury, which is significant for selecting the best treatment[11]. Furthermore, chronic pain also occurs in other neurological conditions such as neuropathy[12]. Finally, in the population with multiple sclerosis, chronic pain can be a significant problem for a substantial number of patients[13]. Therefore, all these conditions cause disability with high direct (medical care, medications) and indirect (loss of productivity) costs[14].

In recent years, social media has become an important source of information where users express and share ideas, opinions, thoughts, and experiences on a multitude of topics[15]. Platforms like Twitter (now X), with over 320 million users, can provide rapid and wide-reaching dissemination of health-related information that can be collected and analyzed for research, including infodemiology and infoveillance[16, 17].

Information obtained through social media is as reliable as traditional survey data[18]. Thus, this information can potentially be useful for understanding specific health conditions15 using data analysis and mining techniques.

The aim of this study is to examine the number and type of tweets (original or retweet) related to chronic pain, as well as to analyze the emotions and compare the societal impact of the diseases under study.

In our hypothesis, we propose that social media can be a space where patients with chronic pain conditions seek help and feel identified and supported by peers and professionals. Additionally, we hypothesize that the interests and considerations regarding chronic pain and its corresponding treatment may differ among patients, family members, experts, and general users.

## Methods

### Study design and data collection

Our cross-sectional study utilized X to gather user posts, in both English and Spanish, regarding diseases associated with chronic pain. The data collection period extended from January 1, 2018, to December 31, 2022.

We used the following keywords (that is, words mentioned in the tweet content): (1) "fibromyalgia"; (2) "headache", "migraine"; (3) "multiple sclerosis"; (4) "polyneuropathy", "neuropathy", "neuralgia"; (5) "paraplegia", "tetraplegia", and their equivalents in Spanish. In total, 72,874 tweets were collected, from each of which we obtained data on the date and time of creation, the publicly displayed username, the text, geolocation, and the status of "likes" and "retweets".

As a data source, we utilized the Tweet Binder API interface, which enabled us to collect all publicly available tweets referencing a series of diseases associated with chronic pain: fibromyalgia, headache, multiple sclerosis, neuropathy, and paraplegia.

The search tool, Tweet Binder[19], allows access to 100% of all public tweets that match the search criteria. Tweet Binder has a data collection system that gathers all publicly available tweets on Twitter and retrieves both the tweets and user information from the Twitter API. Initially, the search tool scans the public section of Twitter to collect tweet IDs that match the search query. Subsequently, a call is made to the Twitter API to retrieve the tweet and user information.

### Sample size

We used LDA, a well-known topic modeling algorithm, which allowed us to ensure good performance. It is particularly well-suited for handling large-scale corpora, similar to the one we have [20–22], as we searched for tweets published over 5 years. Its superior performance in topic determination is noteworthy, as the topics generated are generally more interpretable for humans [23]. Among the various models available, we opted for LDA in this study due to its simplicity, efficiency, and widespread use in contemporary research [24–26].

### Data processing

#### Topic modeling (LDA)

This study adopted an unsupervised learning approach, using Latent Dirichlet Allocation (LDA) for topic modeling. After a comprehensive review of available techniques, LDA was chosen due to its simplicity and widespread utilization, as evidenced in existing work on X [24–26]. Our research primarily focuses on applying a well-documented technique within a novel database, aiming to extract meaningful insights from the data. Before the application of the topic modeling model, an extensive data preprocessing procedure was implemented. This preprocessing encompassed language classification, segregating Spanish tweets from others, and subsequently translating the Spanish tweets into English using the Google translator application. The text was subsequently cleaned by removing stopwords, duplicate words, and nonstandard characters, such as emojis or hashtags.

To find the optimal number of topics in topic modeling, a Cluster Validity Index (CVI) was employed. CVIs are measures used in unsupervised learning to evaluate the effectiveness of clustering by assessing how well data points are organized[27]. The silhouette coefficient was the CVI selected due to its ability to assess both intercluster and intracluster distances. Finally, LDA was applied to the 5 pain-related diseases analyzed (fibromyalgia, headache, paraplegia, neuropathy, and multiple sclerosis).

#### Emotional extraction

Finally, emotion detection was conducted using a model from Hugging Face's machine-learning platform, "Emotional English DistilRoBERTa-base"[28]. This model is recognized as a state-of-the-art model for detecting Ekman's six basic emotions: namely, anger, disgust, fear, joy, sadness, and surprise, with the addition of neutral emotion[29]. Capturing these emotions is crucial in our case, as per the insights from physicians. The emotion detection model demonstrated an accuracy of 66%, surpassing the random baseline probability of 14% (1/7)[24, 29]. This evaluation, conducted by the model's authors, encompassed six different datasets[28, 30]. The model's notable performance establishes its utility and effectiveness for analytical purposes.

## Results

### Number of tweets

For this work, our search tool provided 72,874 original tweets, both in English and Spanish, from January 2018 to December 2022. The number of tweets generated in English was 44,467 (61.02%), while in Spanish, there were 28,407 tweets (38.98%). Of these tweets, 70,588 were analyzed, with the remaining 2,286 tweets considered unclassifiable.

**Fig. 1:**
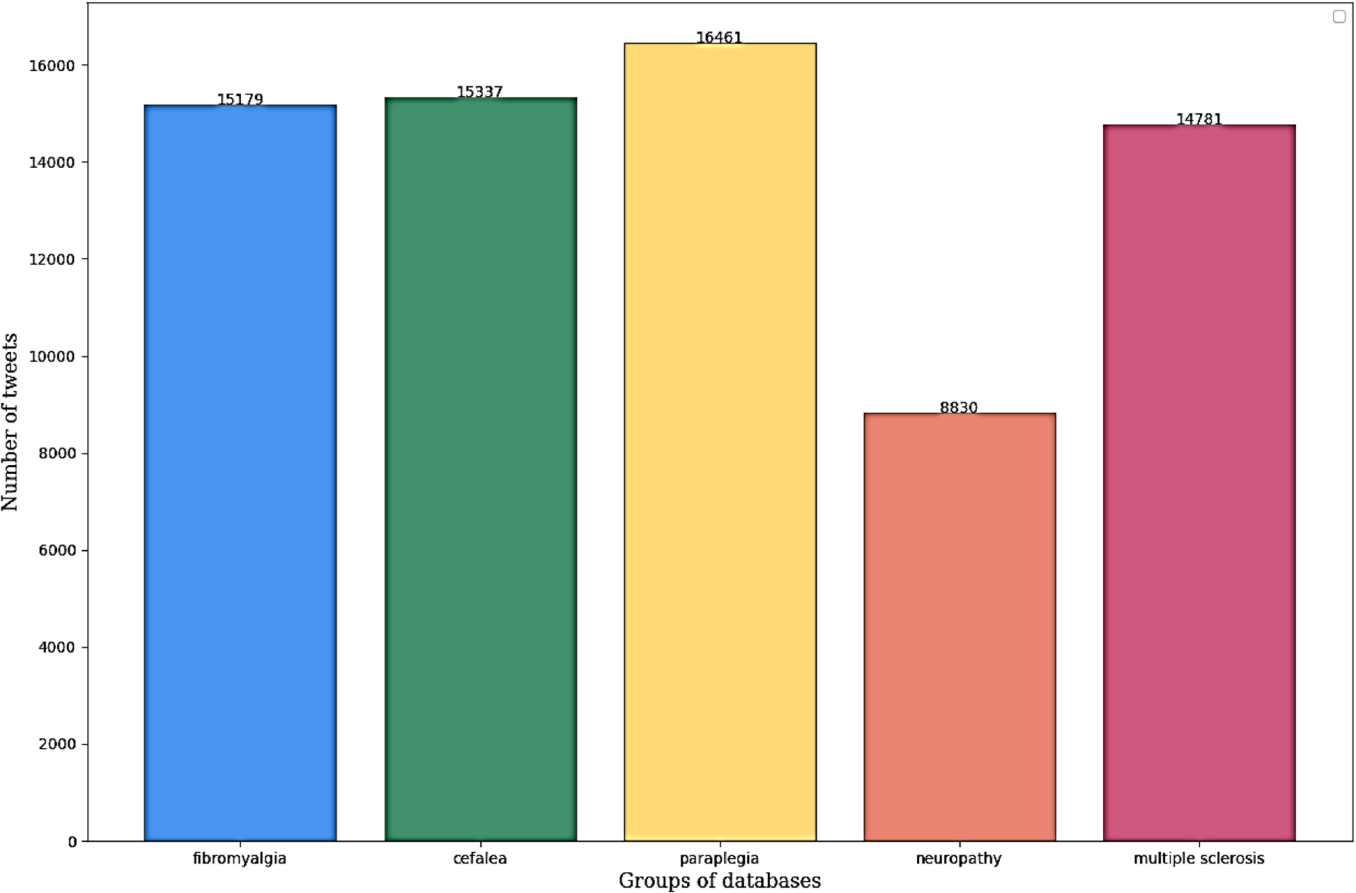
Number of tweets per disease generated by X users between 2018 and 2022

The number of tweets related to each of the diseases (Fig. 1) followed a homogeneous pattern except for the case of neuropathy, which had the lowest frequency of related publications, specifically 8,830 tweets (12.5%). However, there is a preferential accumulation in the case of paraplegia with 16,461 tweets, representing 23.3% of the classifiable tweets. It is followed by headache and fibromyalgia, with similar numbers of tweets, 15,337 (21.7%) and 15,179 (21.5%) tweets, respectively. Finally, 14,781 tweets related to multiple sclerosis represented 21% of the total classifiable tweets.

### LDA

Through LDA, we detected the most frequently associated topics for each disease (Table 1)*.* In the case of headaches, the most recurrent topics are its triggers, the association between headaches and Coronavirus Disease 2019 (COVID-19), and the COVID-19 vaccine and treatments used. Regarding paraplegia, notable topics include advances in research, sporting events, and the etiology of the condition. Focusing on fibromyalgia, topics mainly revolve around the definition of the disease, symptoms, and available treatments. For multiple sclerosis, discussions involve the diagnosis of the disease, efforts to raise funds for research, and new treatments. Finally, when analyzing tweets related to neuropathy, the most prominent topics were the effects of the COVID-19 vaccine, diseases associated with neuralgia, and available treatments.

**Table 1:** LDA Results for each disease

Disease	Topic	Words included
*Headache*	*Topic 1:* Triggers *Topic 2:* Symptoms and side effects of COVID-19 and vaccine for COVID-19 *Topic 3:* Treatments	Vision; Menopause; Caffeine; Insomnia; Bruxism; Screen; Noise; Tobacco; Stress; Depression Fatigue; Fever; Cough; Body aches; Diarrhea; Chills; Joint Pain; Wearyface; Muscle pain; Injection site Cannabis; Marijuana; Yoga; Frozen; Hypnosis; Awareness; Painkillers; Receptor; Genetic; CGRP
*Paraplegia*	*Topic 1: S*cientific research on paraplegia *Topic 2:* Sports events *Topic 3:* Causes of paraplegia	Blood; Tissue; Surgical; Stem Cell; Manage; Neurodegeneration; Genetic; Molecular; Model; Peripheral Foot; Adapt; Free; Film device; Competition; Game; Athlete; Training; Sex; Limb Spinal; Injury; Parkinson; Join; Waist; Traffic accident; Cord; Infection; Vaccine; Neurology
*Fibromyalgia*	*Topic 1:* Definition of the disease *Topic 2:* Symptoms *Topic 3:* Treatments	Chronic illness; Awareness; Invisible illness; Pain; Fatigue; Share; Story; Stiffness; Insomnia; People Memory; Sleep; Muscle; Disturbance; Body; Neuralgia; Point; Leg; Spasms; Sensitivity Cannabis; Exercise; Permanent; Tai chi; Clapping hands; Central; Hypnosis; Debate; Therapy; Emotion
*Multiple sclerosis*	*Topic 1:* Diagnosis *Topic 2:* Donate for research *Topic 3:* Treatments	Brain; Nervous; Lesion; Central; Affect; Spinal; Cord; Symptom; Spasticity; Relapse Donate; Money; Please; Charity; Model; Foldedhands; Beat; Funding; Support; Science Diet; Autoimmunity; Ocrelizumab; Lower; Disease-modifying; Therapy; Injections; Medications; Physiotherapy; Immunomodulators
*Neuropathy*	*Topic 1:* Vaccination *Topic 2:* Diseases related to neuralgia *Topic 3:* Treatments	Shot; Vaccine; Chronic; Inflammation; Single; Immunization; Mass; Postherpetic neuralgia; Mandatory; Antibodies Lupus; Depression; Global; Dysfunction; Mood; Neurological; Herpetic; Seizure; Amyloidosis; Cerebrovascular FDA; Approved; Treatment; Lidocaine; Stage; Topical; Nonopioids; Therapy; Cold; Neurostimulation

Topics detected on twitter and main words of the topic

### Emotional extraction

**Fig. 2:**
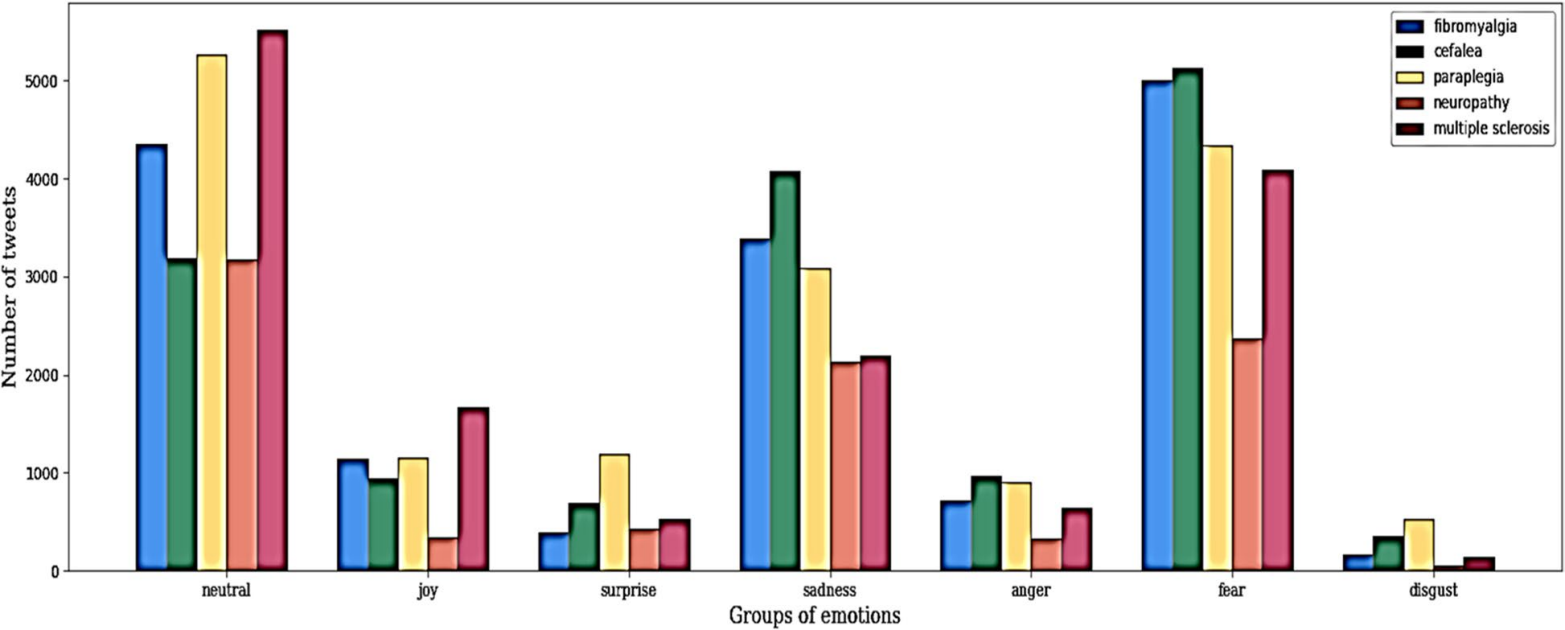
Tweets distribution by disease and emotion between 2018 and 2022

After extracting emotions from the collected tweets, we further investigated the distribution by disease and emotion, attempting to determine the types of emotions (Fig. 2) associated with diseases associated with chronic pain. The results showed that both "fear" and "sadness" were the dominant types of emotions in each group, following the same frequency order according to disease, headache, fibromyalgia, paraplegia, multiple sclerosis, and neuropathy. In contrast, the number of tweets addressing emotions such as "joy," "surprise," "anger," and "disgust" was much lower, with paraplegia, multiple sclerosis, and headache standing out among the diseases.

### Reach and impact

Paraplegia was the content that generated the greatest number of tweets. However, no correlation was detected between the frequency of tweets published in each category and subsequent retweets, with tweets related to headaches showing the highest interest and interaction among users.

**Fig. 3:**
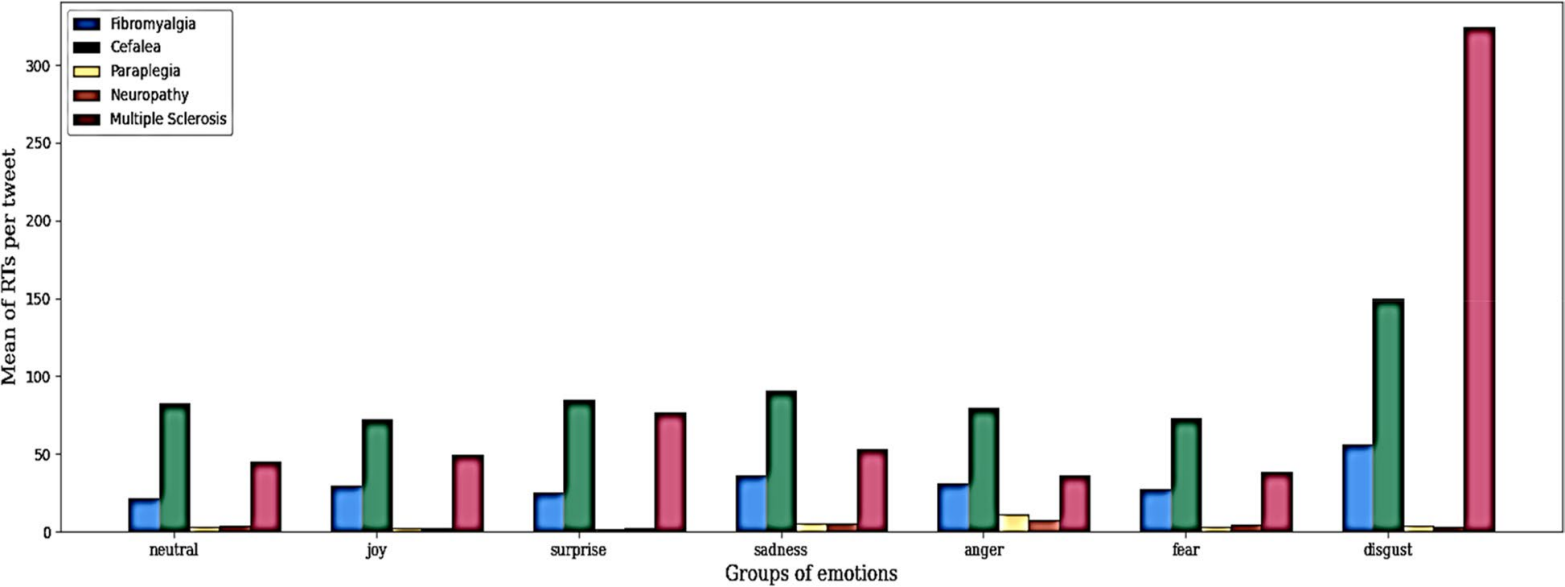
Distribution of the total number of retweets by disease and emotion between 2018 and 2022

We investigated the interest generated by these tweets by examining the number of retweets per disease and emotion (Fig. 3) received. When studying the reach of retweets, the potential impact of tweets addressing headaches associated with emotions such as "joy," "surprise," "sadness," "anger," "fear," and "neutral" was discovered, followed by tweets related to multiple sclerosis and fibromyalgia.

As an exception, tweets expressing a feeling of "disgust" were notable, as they showed a clear difference in the number of retweets related to multiple sclerosis, followed by headache and fibromyalgia.

### Temporal evolution

**Fig. 4:**
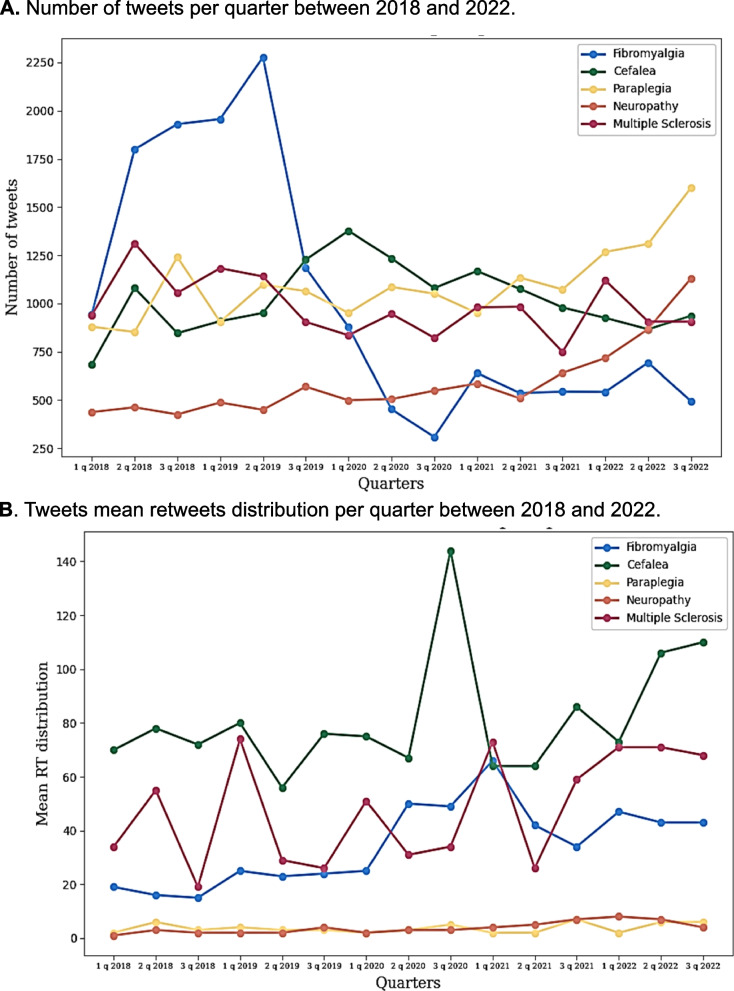
**A** Number of tweets per quarter between 2018 and 2022.** B** Tweets mean retweets distribution per quarter between 2018 and 2022. The data are shown by disease, with each represented by a different color

We evaluated the evolution of the number of tweets published by X users between January 2018 and December 2022 and represented it in a graph over four-month periods (Fig. 4*-A*)*.* Throughout the years analyzed, we observed a progressive increase in the number of tweets generated about fibromyalgia since January 2018, with a particular peak in the second four-month period of 2019. From this point, there was a progressive decrease in the number of publications until the third four-month period of 2020, followed by a plateau in subsequent years, with a new peak occurring from May to August 2022.

Tweets related to paraplegia and neuropathy experienced a similar evolution in the studied five-year period, with a progressive increase in the number of publications starting from the third four-month period of 2021. On the other hand, the trend observed in tweets related to headaches showed several peaks in the second and first four-month periods of 2018 and 2020, respectively. In the publications about multiple sclerosis, the number of tweets followed a homogeneous pattern with several peaks throughout the five years analyzed.

We also studied the kinetics of retweets (Fig. 4*-B*) and observed a homogeneous evolution of tweets related to paraplegia and neuropathy. On the other hand, it is important to note that we observed a particular peak in the number of headache retweets generated in the third four-month period of 2020, followed by a notable increase in subsequent years. However, the evolution of retweets was not homogeneous among the diseases fibromyalgia and multiple sclerosis, where we observed a constant increase or decrease during the years included in our search, especially in the distribution of retweets.

### Geographic location

**Fig. 5:**
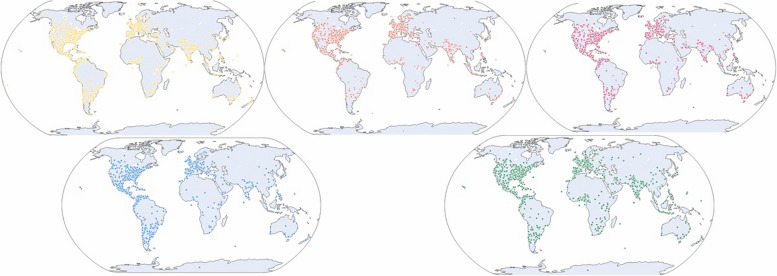
Geolocation Map: Geographic distribution of tweets by disease. Each color represents a different disease (yellow: paraplegia; orange: neuropathy; red: multiple sclerosis; blue: fibromyalgia; green: headache)

We extracted the geographic locations of these tweets to analyze the trends in the volumes of the studied tweets. The distribution of the tweets by continent (Fig. 5) was predominantly from America and Europe rather than from other continents, with their representation being anecdotal.

## Discussion

### Main findings

In this study, based on published tweets, we found that X users were most interested in discussing paraplegia. Through LDA, we found that treatment was the most prevalent topic, followed by etiology and research advancements. Notably, in the case of fibromyalgia, symptoms and the definition of the disease have garnered much attention. The dominant emotions were "fear" and "sadness". Surprisingly, there was no correlation between the number of tweets and their impact measured through generated retweets; ultimately, when analyzing the temporal evolution, the trend in the number of tweets was homogeneous, with a particular peak observed in fibromyalgia-related tweets.

The interest generated by different chronic pain-related diseases among X users was homogeneous, except in the case of neuropathy, where the number of tweets was lower. We know that neuropathy affects older populations more than younger populations, and generally, X users tend to be younger, limiting the information published regarding this condition. On the other hand, the diagnosis of neuropathic pain is underestimated, and these negative or stigmatizing attitudes have led to less interest from society[31]. However, we found studies where such stigmatization further increased the demand for validated information from sources other than formal institutions, more frequently from social media platforms[32, 33]. Conversely, the popularity of paraplegia among X users could have a direct relationship with various clinical and preclinical studies from recent decades reporting on the potential effects of epidural electrical stimulation in the treatment of spinal cord injuries (SCIs)[34], as well as the search for other alternative treatment strategies[35].

Social media plays a significant role in shaping opinions and emotions through the dissemination of information[36], and we believe that retweets serve as a measure of users' particular interest in a topic, which is associated with the emotions evoked by tweets[37]. For instance, sentiment analysis of a large number of messages can provide valuable insights into the mood of the crowd[18] and their health status[38]. The predominance of emotions related to "sadness" and "fear" may be justified because chronic pain is one of the most common health problems in the population, and leads to functional disability, individual suffering, and high costs[39]. These negative emotions also stem from a lack of adequate treatment, as studies in Europe report that approximately 14% of patients discontinued treatment owing to side effects, and up to 40% received treatment they deemed inadequate[40].

In this way, diseases associated with chronic pain are a cause for concern among X users, as reflected in their posts, especially regarding headache, considered the most common neurological disorder in the population[9], and fibromyalgia, both of which have a negative impact on people's well-being[8]. Additionally, the subjectivity in diagnosing both conditions negatively affects public perception of these diseases. Similar to mental illnesses, addiction, and many other painful disorders, migraine is a subjective experience. In comparison to epilepsy, which has a physical manifestation, individuals with chronic migraine are perceived as less reliable, less likely to give their best effort, and more prone to pretending[41, 42]. Consequently, "less visible" diseases or those that evoke greater public rejection are the most feared and cause the most sadness, despite being less severe than others such as paraplegia or multiple sclerosis.

On the other hand, the number of tweets addressing emotions such as "joy" and "surprise" regarding conditions such as paraplegia, multiple sclerosis, and headaches is surprising. This relationship could be explained by recent studies indicating that the European population with SCIs[43] shows high or very high satisfaction with the availability of medical care related to these injuries. In multiple sclerosis, the exact etiology is still unclear, but there has been an association with an abnormal response within the central nervous system, possibly due to an infectious agent[13]. As knowledge about Severe Acute Respiratory Syndrome Coronavirus 2 (SARS-CoV-2) continues to advance, the literature has shown that a significant number of patients experience headaches, the most common and mildest neurological manifestation[44].

In this study, we found that users of X had greater interactions when the content of the tweets was related to headache, likely because headache is the most common neurological disorder in the population[9]. Many studies have demonstrated the use of social media platforms to share experiences with headaches[45, 46]. Additionally, there are multiple reports linking headaches with social media use[8], as somatic symptoms, including headaches, have been found primarily in patients with problematic use of social media[8, 47].

Regarding the temporal evolution of the disease, our data showed that the number of tweets about fibromyalgia decreased between the second and third trimesters of 2019, reaching its lowest level in the third trimester of 2020 in the context of the SARS-CoV-2 pandemic. The uncertainty of the SARS-CoV-2 pandemic caused an abrupt interruption of treatment for patients suffering from chronic pain, resulting in potential unintended harm. For example, in the fibromyalgia population, there is an association between negative emotions ("fear" or "sadness") and less information dissemination, although user interaction is more common. Regarding the effects of the pandemic, studies confirm that COVID-19 has altered the daily functioning of departments by prioritizing hospital services exclusively for life[48]. This not only occurred with fibromyalgia; for instance, research has demonstrated the impact of the pandemic on the treatment of neurological diseases such as multiple sclerosis, especially due to the high risk of initiating immunosuppressive treatments[48, 49] and delays or cancellations of electrodiagnostic studies[48, 50]. On the other hand, there was a general reluctance to visit hospitals among the population. We can speculate that the incidence of neurological emergencies did not decrease during the COVID-19 pandemic but rather that differences in the number of patients were due to alterations in public health policy and users' reluctance to visit hospitals[48]. However, longer-term follow-up studies are needed to evaluate the lasting consequences of the pandemic on the prevalence of neurological diseases and the treatment of patients with chronic neurological disorders[48].

It is relevant to recognize the association of temporal evolution in the analysis of tweets about fibromyalgia and headaches with the SARS-CoV-2 pandemic. The literature describes the persistence of a wide range of symptoms long after the acute phase of severe acute respiratory syndrome caused by SARS-CoV-2[51, 52], including extreme fatigue, musculoskeletal pain, headache, sleep disorders, anxiety, and depression[36, 52, 53]. This clinical picture is associated with poor quality of life and severe deterioration of functional capacity and is known as post-COVID-19 syndrome; these conditions may resemble fibromyalgia because they meet the same diagnostic criteria. Similarly, the exacerbation or onset of fibromyalgia symptoms can occur during or after SARS-CoV-2 infection or due to numerous and persistent stressors imposed daily by the pandemic environment[54]. Awareness among the general population and healthcare professionals about the development of this syndrome may have led to a decrease in interest in fibromyalgia. However, in the same context, publications and interactions about headache increase, because it is part of this syndrome.

### Limitations

This study has several limitations. First, users of X tend to be younger, potentially excluding those segments of the population without internet access or social media (such as the elderly or individuals with low socioeconomic status). This limits the generalizability of our results. Second, we restricted our analysis to tweets in English and Spanish, which may limit the ability to extrapolate the findings. Third, subjectivity can be a significant limitation when specifically coding content that tends to trivialize, where emotional tone or double meaning are very important aspects, as well as negation or irony, which can influence emotional classification. Additionally, the collection of tweets in English and Spanish, followed by their translation, may reduce accuracy in emotional extraction. We also did not differentiate between the number of tweets and the number of users posting these contents, which could lead to inaccuracies in measuring user interest in various diseases.

Other limitations include, on one hand, the choice of diseases in our study, as not all diseases involving chronic pain were included. On the other hand, in our study, we analyzed the geographic location of the users posting the tweets, which may lead to a certain margin of error. Lastly, some tweets may be generated by social media bots.

However, we also highlight that, despite these limitations, our methodology is consistent with previous medical research on Twitter (now X)[50].

## Conclusions

Our results underscore the potential of leveraging social media for a better understanding of patients suffering from chronic pain and its impact on society. Among the most frequently encountered topics are those related to treatment, symptoms, or causes of the disease. Therefore, it is relevant to inform the patient to prevent misconceptions regarding their illness.

### Supplementary Information

Supplementary Material 1.

## Data Availability

The datasets generated and/or analyzed during the current study are available upon reasonable request. In addition, the raw de-identified data may be made available upon reasonable request from the corresponding authors.

## Abbreviations

COVID-19: COronaVIrus Disease 2019
CVI: Cluster Validity Index
IASP: International Association for the Study of Pain
LDA: Latent Dirichlet Allocation
RTs: Retweets
SARS-CoV-2: Severe Acute Respiratory Syndrome COronaVirus 2
SCI: Spinal Cord Injury
